# Does selective logging stress tropical forest invertebrates? Using fat stores to examine sublethal responses in dung beetles

**DOI:** 10.1002/ece3.2488

**Published:** 2016-11-04

**Authors:** Filipe França, Jos Barlow, Bárbara Araújo, Julio Louzada

**Affiliations:** ^1^ Lancaster Environment CentreLancaster UniversityBailriggLancasterUK; ^2^ Departamento de BiologiaUniversidade Federal de LavrasLavras‐MGBrazil; ^3^ Museu Paraense Emilio GoeldiBelém‐PABrazil; ^4^ Laboratório de plantas oleaginosas, óleos, gorduras e biodiesel, Departamento de AgriculturaUniversidade Federal de LavrasLavras‐MGBrazil

**Keywords:** Amazon, conservation physiology, early warning signal, lipid content, physiological stress, reduced‐impact logging, sublethal effects, tropical forest

## Abstract

The increased global demand for tropical timber has driven vast expanses of tropical forests to be selectively logged worldwide. While logging impacts on wildlife are predicted to change species distribution and abundance, the underlying physiological responses are poorly understood. Although there is a growing consensus that selective logging impacts on natural populations start with individual stress‐induced sublethal responses, this literature is dominated by investigations conducted with vertebrates from temperate zones. Moreover, the sublethal effects of human‐induced forest disturbance on tropical invertebrates have never been examined. To help address this knowledge gap, we examined the body fat content and relative abundance of three dung beetle species (Coleoptera: Scarabaeinae) with minimum abundance of 40 individuals within each examined treatment level. These were sampled across 34 plots in a before‐after control‐impact design (BACI) in a timber concession area of the Brazilian Amazon. For the first time, we present evidence of logging‐induced physiological stress responses in tropical invertebrates. Selective logging increased the individual levels of fat storage and reduced the relative abundance of two dung beetle species. Given this qualitative similarity, we support the measurement of body fat content as reliable biomarker to assess stress‐induced sublethal effects on dung beetles. Understanding how environmental modification impacts the wildlife has never been more important. Our novel approach provides new insights into the mechanisms through which forest disturbances impose population‐level impacts on tropical invertebrates.

## 1 Introduction

Humans are causing widespread defaunation of the world as we enter the Anthropocene (Barnosky et al., 2011; Corlett, 2015; Newbold et al., 2014, 2015), a trend expected to continue over the coming years (Pereira et al., 2010). As such, understanding how natural populations respond to environmental modification has never been more pressing. Current research aiming to assess the negative impacts of anthropogenic activities relies chiefly on responses at the species and population levels (Bicknell, Struebig et al., 2014; Newbold et al., 2014).

More recently, there has been increased attention on evaluating the sublethal effects behind the faunal responses to environmental degradation, including fluctuating asymmetry, changes in body conditions, and oxidative levels (Coristine et al., 2014; De Coster et al., 2013; Habel et al., 2014; Rimbach et al., 2013). Growing evidence shows that most environmental degradation impacts on natural populations start with stress‐induced sublethal effects on individuals (Dantzer et al., 2014; Martínez‐Mota et al., 2007). Such sublethal effects are manifested in the physiology, morphology, and behavior of individuals long before responses are expressed and observed at the population scale (Beaulieu & Costantini, 2014; Cooke et al., 2013; Wagner et al., 2013). Therefore, these sublethal responses can act as “early warning signals” to predict future population responses and identify conservation priorities (Dantzer et al., 2014; Hellou, 2011).

Several physiological biomarkers have been frequently used to quantify sublethal stress‐induced responses (Beasley, Bonisoli‐Alquati, & Mousseau, 2013; Beaulieu & Costantini, 2014; Rimbach et al., 2013; Romero, 2004), and studies have shown the importance of downstream metrics (e.g., body fat content and mass changes) to assess the significance and impact of stress in free‐living animals (Breuner, Delehanty, & Boonstra, 2013; Knapp, 2016). For example, body fat content has been frequently used as a physiological biomarker to assess sublethal effects of many environmental stressors on vertebrates (Lucas et al., 2006; Suorsa et al., 2003, 2004) and insects (Irschick et al., 2013; Piiroinen et al., 2014; Reaney & Knell, 2015). Overall, such studies have shown that organisms under stressful conditions exhibit changes in the fat storage (Baldal et al., 2005; Piiroinen et al., 2014), which is suggested as a strategy to increase life span as a result of improved energy consumption efficiency (Broughton et al., 2005; Hansen, Flatt, & Aguilaniu, 2013) and to improve the starvation stress resistance (Arrese & Soulages, 2010; Lee & Jang, 2014). However, changes in the fat metabolism can have negative effects on individual physiological balance (Moghadam et al., 2015; Van Praet et al., 2014), which might reduce the organism fecundity and reproduction (Arrese & Soulages, 2010; Barry & Wilder, 2013; Hansen et al., 2013).

Despite progress made in our understanding of how forest degradation can induce sublethal effects on wildlife, two key areas remain underexplored in the literature. First, there is a clear bias toward temperate regions (Leshyk et al., 2012; Mastromonaco, Gunn, & Edwards, 2014; Suorsa et al., 2003, 2004; but see Rimbach et al., 2013), yet extinction rates in the tropics are alarming (Laurance, 2007; Pereira et al., 2010; Pimm et al., 2014), as species are disappearing before being discovered (Lees & Pimm, 2015). Thus, our knowledge of forest degradation sublethal effects on the hyperdiverse and highly threatened tropical fauna is very limited. Secondly, where studies have been conducted in the tropics, they have typically investigated how forest degradation influences vertebrates (e.g., Mastromonaco et al., 2014; Rimbach et al., 2013; Suorsa et al., 2003, 2004) and evidence of how human disturbances induce sublethal effects on tropical invertebrates is, to our knowledge, lacking in the literature.

**Figure 1:**
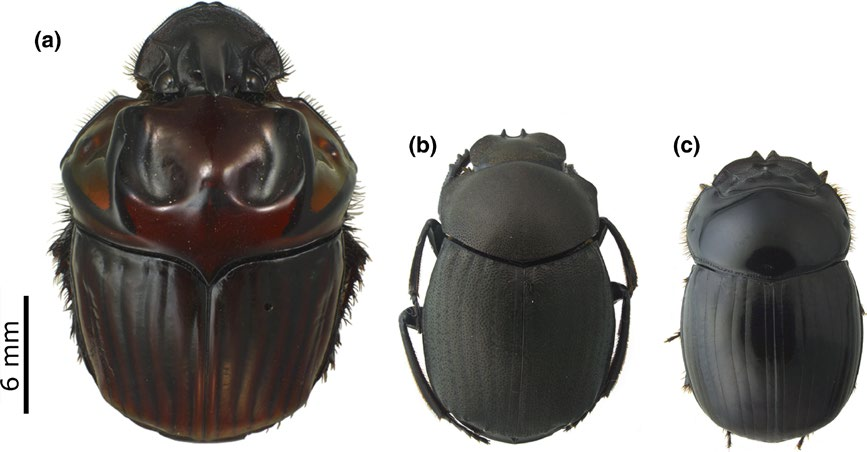
(a) *Oxysternon festivum*, (b) *Deltochilum* aff. su*bmetallicum*, and (c) *Dichotomius lucasi* are dung beetle species (Coleoptera: Scarabaeinae) found in the Brazilian Amazon. Photographs are scaled to each other; the largest species is (a) *O. festivum* (length: 20.6 mm); the smallest species are (b) *D*. aff. su*bmetallicum* (length: 13.1 mm) and (b) *D. lucasi* (length: 11.5 mm). Photographs taken by A.P. de Arcanjo

To help address this knowledge gap, we investigate the sublethal and population‐scale impacts of selective logging on dung beetles (Coleoptera: Scarabaeinae; see Figure 1). Selective logging is considered a major disturbance driver throughout tropical forests (Asner et al., 2009; Blaser et al., 2011) and can induce sublethal effects in vertebrates (Lucas et al., 2006; Rimbach et al., 2013; Suorsa et al., 2003, 2004). We considered the fat content of three abundant dung beetle species as a biomarker for logging sublethal effects and compared it to their relative abundance, which reflects their population‐scale response to logging. Dung beetles are an appropriate focal insect group that are cost‐effective to sample (Gardner et al., 2008) and have been widely used to assess the selective logging impacts on biodiversity (Bicknell, Phelps et al., 2014; Davis et al., 2001; Edwards et al., 2012; França et al., 2016). We hypothesized that body fat content of dung beetles in selectively logged forests would increase as a sublethal response to new stressful environmental conditions. Furthermore, we also examine whether these stress‐induced sublethal effects on the same species would match with their relative abundance responses to logging.

## 2 Materials and Methods

### 2.1 Study area and sampling design

Beetles were sampled within the 1.7 Mha *Jari Florestal* landholding, located in the northeastern Brazilian Amazonia (00°27′–01°30′ S, 51°40′–53°20′ W; Figure S1 in Appendix S1). The area comprises a mosaic of Eucalyptus plantations and regenerating secondary forests within ~1.5 Mha of primary forests subjected to very low levels of disturbance (Barlow et al., 2010; Parry, Barlow, & Peres, 2009). Forests in this region are characterized as evergreen dense tropical rainforest (Souza, 2009), with a high density of the timber species *Dinizia excelsa* Ducke (Fabaceae, Mimosoideae) (Laufer, Michalski, & Peres, 2015), which corresponds to about 50% of exploited timber in some Amazonian regions (Barbosa, 1990).

Reduced‐impact logging (RIL) started in 2003, with plans to log approximately 544,000 ha of native forest over a 30‐year cutting cycle. This management produces ~30,000 m^3^ of timber annually and is one of the largest Amazon logging concessions under the Forest Stewardship Council certification (FSC 2014). Logging operations follow FAO guidelines (Dykstra & Heinrich, 1996) to minimize its impacts via a preharvest inventory, which maps, measures, and identifies all commercially viable trees with d.b.h. ≥ 45 cm. Therefore, the skid trails and logging road network is minimized, tangling lianas are cut before harvesting, and directional felling is used to reduce collateral damage to other trees. After logging operations in 2012, the timber removal average within the harvested area was about 16.8 m^3^/ha, which is within RIL protocols that set an upper limit from 25 m^3^/ha for the 30‐year cutting cycle (IFT, 2014, CONAMA Resolution 406/2006).

We used a BACI experimental design to sample dung beetles at 34 independent sites of 10 ha (250 × 400 m) across this logging concession. Five of these sites were primary forest controls, which remained unlogged during the course of the study, and were about 6.5 km from the closest logged sites. The other 29 sites were logged under a gradient of logging intensity from 0 to 50.31 m^3^/ha of extracted timber (or 0–7.9 trees/ha).

Two dung beetle surveys were conducted at exactly the same locations and using the same methods at all 34 sites. The first survey (prelogging) was conducted in June and July 2012, approximately 45 days before the logging operation began. The second survey (postlogging) took place in 2013, around 10 months after the logging activities ended. It also occurred in June and July, to minimize possible effects from seasonal variation. Sampling locations were relocated based on marking tape, or by GPS when disturbance from logging activities meant this could not be found. Community‐level analysis shows there was low degree of spatial autocorrelation in prelogging biodiversity data, which disappeared in the postlogging survey (França et al., 2016).

### 2.2 Dung beetle collection

Dung beetles were collected with baited pitfall traps, which were plastic containers (19 cm diameter and 11 cm deep) buried in the ground with their opening at ground level, containing approximately 250 ml of a saline solution and a plastic lid as a rain cover. A small plastic cup containing approximately 35 g of pig dung mixed with human dung (4:1 pig‐to‐human ratio, according to Marsh et al., 2013) was attached by a wire above each pitfall. All dung beetles that fell in pitfall traps were dried and transported to the laboratory where they were identified to species level, or morphospecies level where this was not possible. Voucher specimens were added to the Reference Collection of Neotropical Scarabaeinae in the Insect Ecology and Conservation Laboratory, Universidade Federal de Lavras, Brazil.

### 2.3 Samples preparation and body fat extraction

To examine whether selective logging induces stress sublethal responses on invertebrates, we assessed the body fat content of the three abundant dung beetle species *Oxysternon festivum* Linnaeus, 1767, *Dichotomius* (Luederwaldtinia) *lucasi* (Harold, 1869), and *Deltochilum* (Deltohyboma) aff. *submetallicum* (Castelnau, 1840). For analysis purpose, we selected these species based on the minimum abundance of 40 individuals within each treatment level (control and logging sites) for each survey event (prelogging and postlogging).

Before fat extraction, all examined individuals were oven‐dried at 40°C for 48 h and dry‐weighed with a *Shimatzu* AY220 balance scale (*Shimadzu Corporation, Kyoto, Japan*) accurate to within ±0.0001 g. Dried and weighed individuals were assigned an identification number and placed into a labeled extraction thimble (~30 mm width and 60 mm length) made from thick filter paper. In order to increase the measurement efficacy of fat storage of the smaller species, we placed two individuals of *D*. aff. *submetallicum* or *D. lucasi* (*N* = 156 and 160 individuals in total, respectively) into each extraction thimble. The average body fat content of two individuals of each species was then measured as an extraction sample. For the larger *O. festivum* species, we individually weighed 80 beetles before placing each one into an extraction thimble.

All extraction thimbles were loaded into the main chamber of the Soxhlet extractor apparatus (*MA 487/6/250 Marconi Equipamentos para Laboratórios, Piracicaba, Brazil*), which was fitted to a tared distillation flask containing five boiling glass regulators. Body fat content was extracted using 200 ml of *n*‐hexane heated under reflux for 4 hr (18–22 cycles/hr) at 63–65°C. We opted to use Soxhlet fat extraction as it has been suggested as a reference for evaluation of other extraction methods (Tzompa‐Sosa et al., 2014; Wang et al., 2010) and has been previously used to assess the body fat content of dung beetles and other insects (Edwards, Division, & Box, 1988; Hart & Tschinkel, 2012; Moya‐Laraño et al., 2008; Tzompa‐Sosa et al., 2014).

After extraction, all thimbles were left in a walk‐in fume cupboard (*SP‐Labor, model SP‐150N, average duct velocity = 60 m* ^*3*^ */min, São Paulo, Brazil*) for 24 hr to eliminate the major excess of solvent. Subsequently, thimbles were oven‐dried for another 48 hr at 40°C to assess the fat‐free weight. Total fat content (g) was measured by subtracting the fat‐free weight from the dry‐weight for each beetle (or pair of beetles). In thimbles that contained two individuals, we considered the average weight difference as the measure of body fat content.

### 2.4 Statistical analyses

All statistical analyses were performed using the R software (R Core Team 2015). We used generalized linear models (GLMs) with a logarithmic link function (Zuur et al., 2009) in the *glm()* routine (*stats* package, R Core Team 2015) to assess the effects of the explanatory variables “time” (two levels: prelogging and postlogging) and “treatment” (two levels: control and logging sites) on the response variables dung beetle body fat content and relative abundance. Because dung beetle are highly sensitive to changes in environmental conditions (Bicknell, Phelps et al., 2014; Menéndez et al., 2014), we also used GLMs to check for assessing changes in the canopy openness (see Appendix S2). We ran an independent GLM followed by a two‐way ANOVA including the factors “time,” “treatment,” and the interaction “time:treatment” for each dung beetle species and response variable. Post hoc *t*‐test pairwise comparisons were conducted when both factors significantly affected the response variables. All graphs depict untransformed data.

We used the *shapiro.test* function (*stats* package, R Core Team 2015) to perform Shapiro–Wilk tests of normality for response variables and model residuals. Where appropriate, quasi‐Poisson or quasi‐binomial likelihood GLMs were performed as recommended to deal with overdispersed count data (Ver Hoef & Boveng, 2007). Quantile plots and plots of residuals with predicted values versus standardized deviance values were also inspected to ensure that homogeneity and error distributions were appropriate (Crawley, 2013). Lastly, in order to provide an intuitive visual assessment of any spatial effects in the analysis (Baddeley et al., 2005; Kühn & Dormann, 2012), we plotted the residuals from GLMs with the relative abundance of each species on spatial maps of the sample sites (see Appendix S2 for details).

When measuring the body fat content, we assessed two dependent variables: dung beetle biomass (dry‐weight prior fat extraction) and body fat content (difference among dry and fat‐free weight). Because we aimed to examine changes in the dung beetle body fat content, we included body mass values as an offset term in the GLMs (Zuur et al., 2009). An offset includes a known term to the linear model without fitting its parameters (Crawley, 2013). This approach has been recommended to handle ratios without missing the discrete nature of the response, or when collinearity may be expected (Bolker, 2012). Thus, we modeled the effects of selective logging on the ratio between the body fat content and body biomass, offsetting the confounded effect of biomass. Comparisons between changes in the canopy openness, changes in relative abundance, and changes in body fat content of each species were made qualitatively.

## 3 Results

### 3.1 Sublethal effects of selective logging

**Figure 2:**
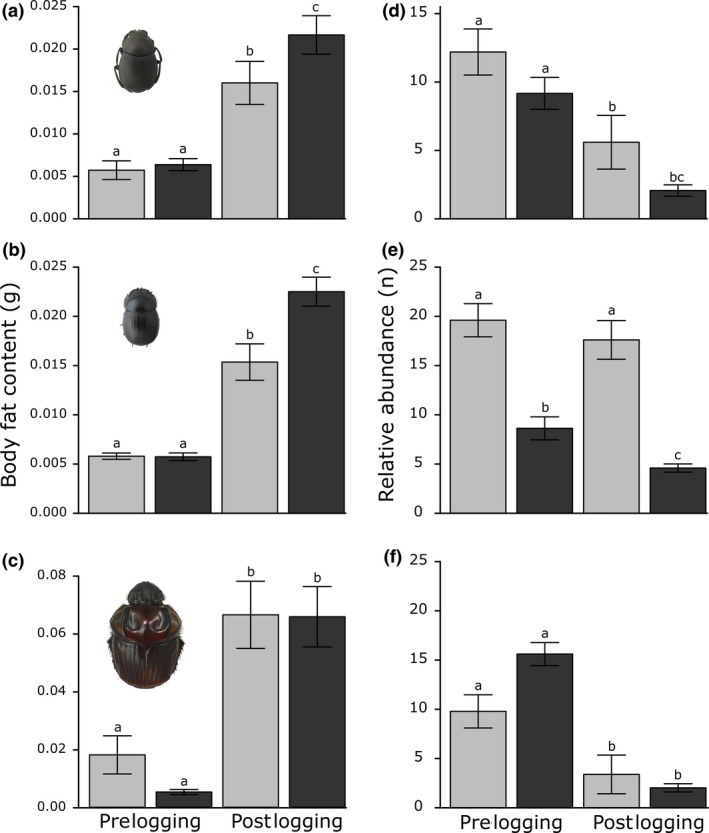
Sublethal and relative abundance effects of selective logging on the dung beetle species *Deltochilum* aff. *submetallicum* (a, d); *Dichotomius lucasi* (b, e); and *Oxysternon festivum* (c, f). On the left panels, sublethal effects were measured on the fat body content (g) and right panels show the relative abundance of each dung beetle species. Because we used a BACI experimental design, left bars from each panel show the prelogging data and right bars represent postlogging. Data from control sites are present in light gray bars and from logging sites are present in dark gray bars. Photographs are scaled to each other. Means ± standard error of the mean (SEM) followed by different lowercase letters indicate significant differences with an alpha of .05, based on post hoc *t*‐test pairwise comparisons

There were significant differences in the body fat content sampled between the treatments over the course of the study for all examined dung beetle species (Figure 2a–c). Irrespective of logging treatment, all dung beetle species increased their fat content in the second survey (Figure 2a–c). However, significant logging‐induced sublethal effects were found for *D*. aff. *submetallicum* and *D. lucasi* species, which presented greater fat content at the selectively logged sites than in the unlogged control sites (two‐way ANOVA: *D*. aff. *submetallicum* treatment × time interaction *F*_3,74_ = 8.81, *p* = .004; *D. lucasi* treatment *F*_1,78_ = 15.56 & time *F*_2,77_ = 15.56, all *p* < .001; *O. festivum* time *F*_1,78_ = 16.30, *p* < .001). Post hoc analysis supported these findings for the species *D*. aff. *submetallicum* and *D. lucasi*, as individuals had higher body fat content at logged sites compared with unlogged sites (*t*‐test, *p*‐values ≤.001; Table S1 in Appendix S1).

### 3.2 Population‐level effects of selective logging

Changes in dung beetle relative abundances were qualitatively similar to the sublethal responses of each species (Figure 2d–f). Although dung beetle relative abundances differed among treatment levels for all species (two‐way ANOVA: *D*. aff. *submetallicum* treatment *F*_1,66_ = 4.05, *p* = .04 & time *F*_2,65_ = 44.02, *p* < .001; *D. lucasi* treatment *F*_1,66_ = 29.62 & time *F*_2,65_ = 7.31, all *p* < .008; *O. festivum* time *F*_1,66_ = 80.64, *p* < .001), consistent negative logging‐induced impacts were found just in the relative abundance of the *D. lucasi* species (*t*‐test, *p*‐value <.001; Table S1 in Appendix S1). Finally, there was no discernible visual association between model residuals from relative abundance and geographical location (Fig. S2 in Appendix S1).

## 4 Discussion

Our results demonstrate that selective logging can generate sublethal responses in dung beetle condition, supporting the notion that forest disturbances can affect invertebrates’ physiology. Moreover, these sublethal responses were qualitatively similar to the changes in dung beetles’ relative abundance (Figure 2a–c), which supports the measurement of body fat as a potential biomarker of insect stress.

### 4.1 Sublethal effects of selective logging

Most energy reserves of insects are stored in the body fat (Arrese & Soulages, 2010), which regulates physiological and behavioral responses to environmental and human‐induced conditions (Arrese & Soulages, 2010; Edwards et al., 1988; Reaney & Knell, 2015). As many studies have indicated the increased fattening of insects in response to other stress‐induced conditions (Hansen et al., 2013; Moghadam et al., 2015; Piiroinen et al., 2014), we suggest that the increased body fat content in dung beetles from logged forests reinforces the use of this trait as a likely early warning biomarker to assess the environmental quality of disturbed tropical forests.

Several lines of evidence indicate that stressful conditions (e.g., future food shortages or periods of high‐energy demand) may induce fat accumulation as a strategy to conserve energy for processes needed for survival (Broughton et al., 2005; Hansen et al., 2013; Schneider, 2014). It may be that the postlogging environmental conditions, such as the increased canopy openness we found in the second survey at logged sites (Figure S3 in Appendix S1) and the higher temperatures at forest gaps from logged forests (Asner, Keller, Pereira, et al. 2004; Asner, Keller & Silva 2004; Forrester et al., 2012), are likely responsible for the observed dung beetle fattening.

Another possible explanation is that the observed fattening in dung beetles could be related to a reduction in reproduction rates. Reproduction is a high cost process (Hansen et al., 2013), and fat content is considered the currency (Barry & Wilder, 2013). Although not always observed (Flatt et al., 2008; Wilder, Raubenheimer, & Simpson, 2015), the link between reduced reproduction and increased fat storage has been demonstrated for many insects (Hansen et al., 2013; Moghadam et al., 2015; Wilder et al., 2015). Similarly, previous research has found a positive relationship between reduced fecundity, increased fattening, and higher starvation stress resistance (Broughton et al., 2005; Hansen et al., 2013). These findings reinforce that under stressful conditions, invertebrates may modify their fat metabolism as a strategy to optimize energy allocation for surviving likely starvation periods (Hansen et al., 2013).

It remains uncertain how increased body fat content will influence long‐term patterns in dung beetle populations. Having a high proportion of body fat does not necessarily translate to high reproductive success when insects are deficient in other nutrients necessary for maintaining a healthy metabolic function or for effective reproduction (Barry & Wilder, 2013; Wilder et al., 2015). As organisms relocate part of energy reserves to repair or maintain their physiological integrity under stressful environments, less energy is left for individual growth and eventual reproduction (Van Praet et al., 2014). This could lead to further reductions in population sizes and may explain the qualitative connection between sublethal effects and the relative abundance we found for the three assessed dung beetle species.

### 4.2 Interannual variation and species‐specific responses

Although we found that body fat content was higher in individuals of two species (*D*. aff. *submetallicum* and *D. lucasi*) sampled at logged sites, body fat content also increased, irrespectively of where, in the second survey for all species. This result was somewhat surprising, because dung beetle collections were carried out in the same months (June and July) in both years to minimize seasonal effects. Nevertheless, it may reflect the seasonal variation of environments, such the increased canopy openness we found in the control sites in the second survey (Figure S3 in Appendix S1), leading to changes in biomarkers related to survival and reproduction (i.e., fat content and body mass), as described for moths (Davidowitz & Nijhout, 2004), dung beetles (Edwards et al., 1988), and carabid beetles (Östman, 2005).

Studies have shown that different species may cope differently with forest fragmentation and selective logging (Leshyk et al., 2012; Lucas et al., 2006; Mastromonaco et al., 2014; Suorsa et al., 2003, 2004; Wasser et al., 1997). Thus, we highlight that not all species may have predictable and similar physiological responses to forest disturbances (as suggested by Rimbach et al., 2013), even when consequences in relative abundance are negative for most of them. Moreover, it is very likely that physiological plasticity may also be sex‐linked and age‐dependent (Knapp, 2016; Piiroinen et al., 2014; Stillwell & Davidowitz, 2010; Zhang et al., 2010). However, the low sample size in disturbed postlogging sites prevented us from separating dung beetles by gender and to make more inferences about reproduction aspects and sex‐specific responses.

Our study highlights two important areas for further research: (1) To examine fat stores and maintain statistically meaningful sample sizes, we only examined body fat in the three species that were abundant across all treatment levels. Studies with greater spatial replication and temporal resolution would enable authors to examine whether body fat predicts the extirpation of the most disturbance‐sensitive species. (2) We examined just one type of forest disturbance influencing just the fat storage from an insect group, and it is not clear how different forest disturbances induce sublethal responses on distinct invertebrates, or whether different invertebrate groups respond similarly. Addressing these points may increase our ability to better understand the consequences of forest disturbances on tropical fauna, thus enabling us to improve conservation strategies (Coristine et al., 2014).

## 5 Conclusion

Understanding how impacts of environmental degradation on natural populations are driven by physiological mechanisms has never been more critical (Busch & Hayward, 2009). Our results reveal that selective logging brings about sublethal effects indicative of stress in tropical forest invertebrates. Therefore, we are confident that body fat content is a reliable biomarker to assess stress in dung beetles that persist in disturbed environments. Our study therefore reinforces the potential role of physiological biomarkers as a tool to improve our current ability to make predictions about the future impacts of forest degradation on wildlife (Coristine et al., 2014; Dantzer et al., 2014; Ohlberger, 2013). Lastly, we highlight future research that would help elucidate the relative importance of environmental degradation and forest disturbances in driving stress‐induced physiological impacts on tropical invertebrates.

## Conflict of Interest

None declared.

## Data Accessibility

All data used in this manuscript are present in the supporting information.
