# Supplementary material for: Does selective logging stress tropical forest invertebrates? Using fat stores to examine sublethal responses in dung beetles
Source: Ecol Evol. 2016 Nov 4;6(23):8526–33. doi: 10.1002/ece3.2488 (PMC5167030; doi:10.1002/ece3.2488)
Supplement: Supplementary file 1 [file ECE3-6-8526-s001.doc]

**Appendix S1.** Supporting tables and figures.

**Figure S1.** Map of study area. (A) Brazil; (B) state of Pará; (C) *Jari* landholding and (D) the experimental design in *Jari* region where we sampled dung beetles within 34 planned logging units. The units that we sampled and were selectively logged after the first dung beetle collection are highlighted in dark grey (1-29), whereas the five control units, which remained unlogged during the course of the study, are clear (30-34).


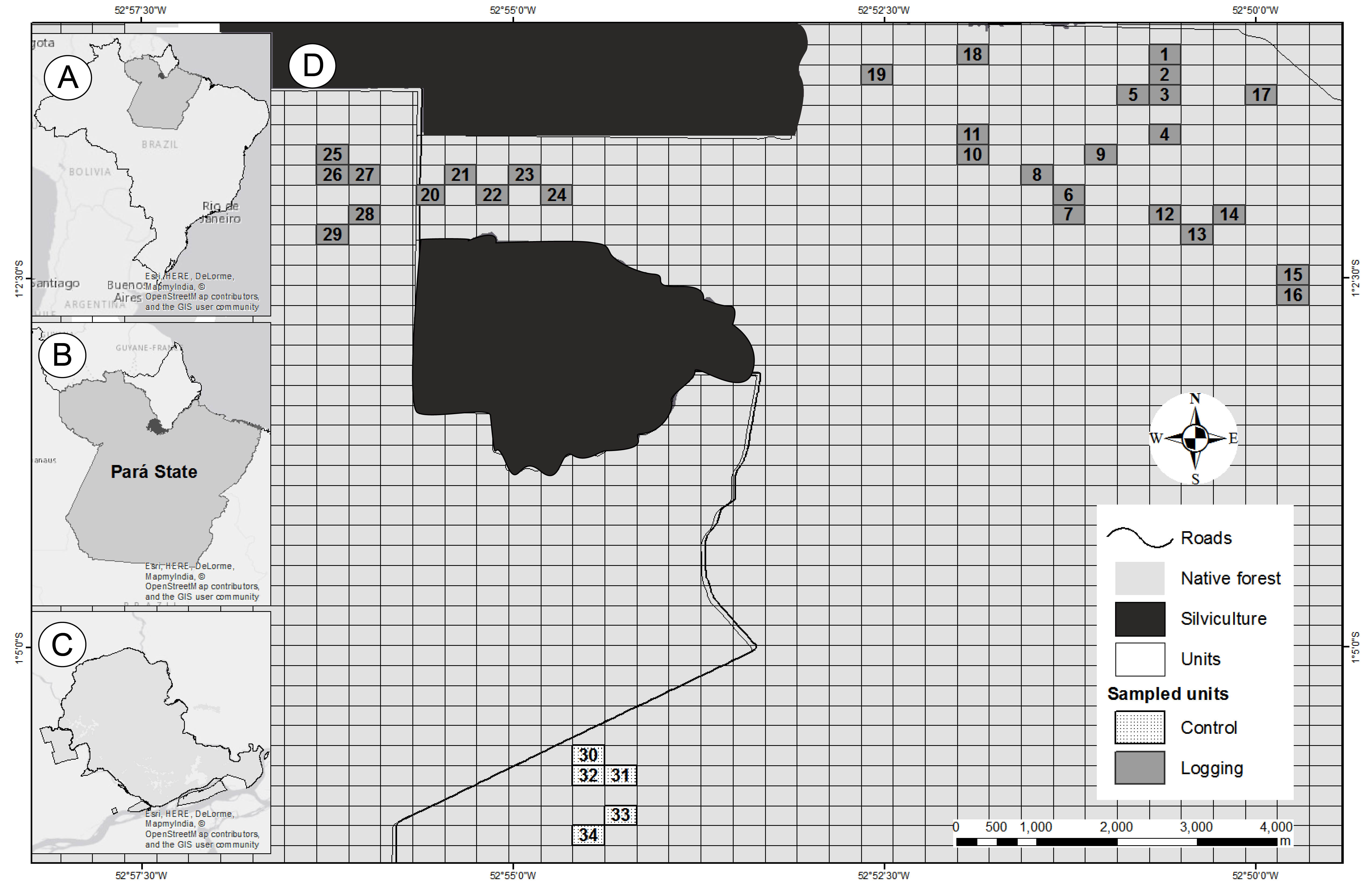


**Figure S2.** Map of residuals from linear models with the relative abundance of the examined dung beetle species in the Amazon forest, Brazil. Radii proportional to the absolute value of residuals. Black dots represent the 29 logging units with different selective-logging intensities and the five grey dots represent the five unlogged control units.


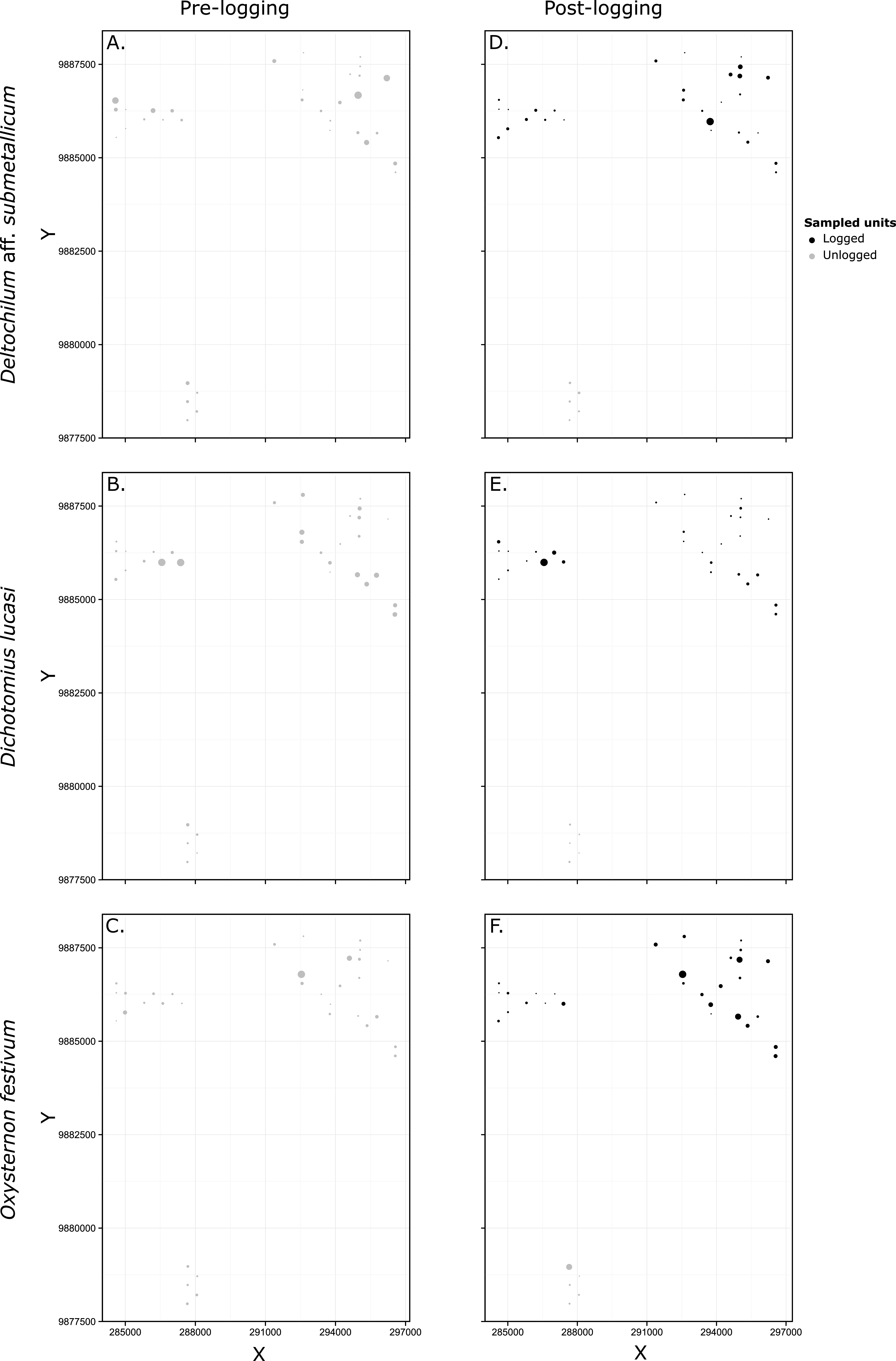


**Figure S3.** Differences between control (dark grey bars) and logging sites (light grey bars) in surveys performed before (left bars in the panels) and after selective-logging (right bars in the panels) for canopy openness. Means ± standard error of the mean (SEM) followed by different lower-case letters indicate significant differences with an alpha of 0.05, based on post hoc t-test pairwise comparisons (see Table S2).


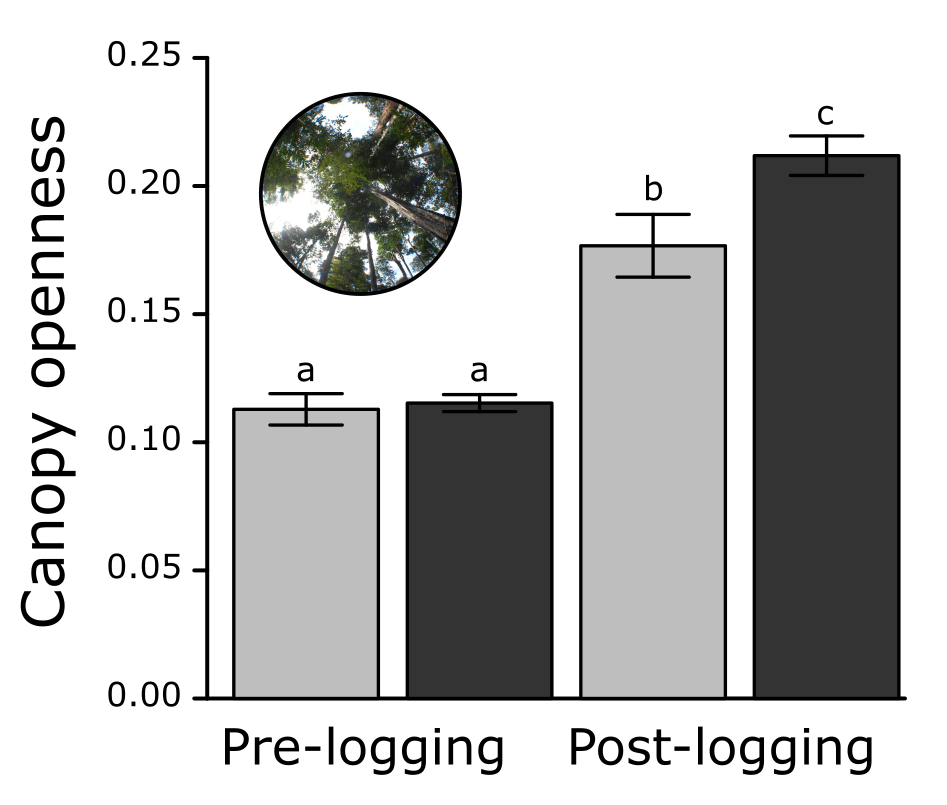


**Table S1.** Post hoc t-test pairwise comparisons for GLM models including the body fat content and relative abundance of the examined dung beetle species: *Dichotomius lucasi* (Harold, 1869); *Oxysternon festivum* Linnaeus, 1767; and *Deltochilum* aff. *submetallicum* (Castelnau, 1840). Pairwise comparisons were conducted with the *pairwise.t.test* function from stats package (R Core Team 2015).

|  |  | **Fat content** | | | **Abundance** | | |
| --- | --- | --- | --- | --- | --- | --- | --- |
|  |  | Pre-control | Pre-logging | Post-control | Pre-control | Pre-logging | Post-control |
| *Deltochillum* aff *submetallicum* | Pre-logging | 0.64 | - | - | 0.18 | - | - |
|  | Post-control | <0.001 | <0.001 | - | 0.02 | 0.11 | - |
|  | Post-logging | <0.001 | <0.001 | <0.001 | <0.001 | <0.001 | 0.12 |
| *Dichotomius lucasi* | Pre-logging | 0.97 | - | - | <0.001 | - | - |
|  | Post-control | <0.001 | <0.001 | - | 0.56 | 0.0012 | - |
|  | Post-logging | <0.001 | <0.001 | <0.001 | <0.001 | 0.0065 | <0.001 |

**Table S2.** Post hoc t-test pairwise comparisons for GLM models using the canopy openness as response variable. Pairwise comparisons were conducted with the *pairwise.t.test* function from stats package (R Core Team 2015).

|  | **Canopy openness** | | |
| --- | --- | --- | --- |
|  | Pre-control | Pre-logging | Post-control |
| Pre-logging | 0.87 | - | - |
| Post-control | 0.001 | <0.001 | - |
| Post-logging | <0.001 | <0.001 | 0.021 |
