# Supplementary material for: Does selective logging stress tropical forest invertebrates? Using fat stores to examine sublethal responses in dung beetles
Source: Ecol Evol. 2016 Nov 4;6(23):8526–33. doi: 10.1002/ece3.2488 (PMC5167030; doi:10.1002/ece3.2488)
Supplement: Supplementary file 2 [file ECE3-6-8526-s002.doc]

**Appendix S2.** Supplementary methods.

**Spatial residual plots:**

We used maps of residuals (spatial residual plots) to provide an effective diagnose of spatial trends influencing model results (Baddeley et al. 2005; Kühn & Dormann 2012). Thus, we mapped the spatial distribution of residuals from each linear model with dung beetle relative abundance as response variable, and used the function ‘residuals.lm()’ from ‘stats’ package (R Core Team 2015) to obtain the residuals of each sampled unit from all fitted linear models. Then, the residuals of these models were plotted against its spatial location, wherein the magnitude of each point represents the absolute value of residuals for each sampled unit. The plots do not reveal any consistent influence of spatial location on model residuals (see Fig. S2 in Appendix S1).

**Canopy openness differences:**

Canopy openness was quantified by taking hemispherical photographs with a Nikon FC-E8 fisheye lens attached to a Nikon D40 camera levelled ~1.20. Photographs were taken when the sky was overcast or in early morning and late afternoon using optimum exposure for each site (Zhang et al. 2005). The Gap Light Analyser software (GLA version 2.0; Frazer et al., 1999) was used to estimate the ‘% canopy openness’ factor, which represents the ratio of the total amount of open space to the total area of the hemispherical photograph (Frazer et al. 1999). This approach has been widely used to account for the canopy openness (Gries et al. 2012; Medjibe et al. 2014; Niemczyk et al. 2015; Silveira et al. 2010). Canopy openness significantly changed between pre- and post-logging surveys, thereby, having higher values in logged sites than in control sites in the second survey (time F_2,65_ = 1677.98, *P* < 0.001 & treatment F_1,66_ = 3.57, *P* = 0.06; see Fig S3 in Appendix S1).

**Supplemental references**

Baddeley, A. et al., 2005. Residual analysis for spatial point processes. *Journal of the Royal Statistical Society Series B-Statistical Methodology*, 67, pp.617–651. Available at: <Go to ISI>://000233203400001.

Frazer, G., Canham, C. & Lertzman, K., 1999. Gap Light Analyzer (GLA), Version 2.0: Imaging software to extract canopy structure and gap light transmission indices from true-colour fisheye photographs, users manual and program documentation. *Program*, p.36.

Gries, R. et al., 2012. Evaluating the impacts and conservation value of exotic and native tree afforestation in Cerrado grasslands using dung beetles. *Insect Conservation and Diversity*, 5(3), pp.175–185. Available at: http://doi.wiley.com/10.1111/j.1752-4598.2011.00145.x.

Kühn, I. & Dormann, C.F., 2012. Less than eight ( and a half ) mis- conceptions of spatial analysis. *Journal of Biogeography*, 39, pp.995–998.

Medjibe, V.P. et al., 2014. Natural regeneration of selected timber species in the Republic of Congo. *African Journal of Ecology*, 52(4), pp.552–563. Available at: http://doi.wiley.com/10.1111/aje.12167.

Niemczyk, M., Żółciak, A. & Piotr, W., 2015. The influence of stand canopy openness on the growth of common yew (Taxus baccata L.). *Forest Research Papers*, 76(1), pp.42–48. Available at: http://www.degruyter.com/view/j/frp.2015.76.issue-1/frp-2015-0004/frp-2015-0004.xml.

R Core Team, 2015. R: A language and environment for statistical computing. *R Foundation for Statistical Computing, Vienna, Austria.* Available at: http://www.r-project.org/.

Silveira, J.M. et al., 2010. Factors Affecting the Abundance of Leaf-Litter Arthropods in Unburned and Thrice-Burned Seasonally-Dry Amazonian Forests A. Hector, ed. *PLoS ONE*, 5(9), p.e12877. Available at: http://dx.plos.org/10.1371/journal.pone.0012877.

Zhang, Y., Chen, J.M. & Miller, J.R., 2005. Determining digital hemispherical photograph exposure for leaf area index estimation. *Agricultural and Forest Meteorology*, 133(1-4), pp.166–181. Available at: internal-pdf:/Zhang et al 2005 Determining digital hemispherical photograph exposure for leaf area index estimation.pdf\nhttp://www.sciencedirect.com/science/article/B6V8W-4HCMS5J-1/2/0be9a14b203bbb8e91ff1241a50b021a.
